# Relationship between Respiratory Function and the Strength of the Abdominal Trunk Muscles Including the Diaphragm in Middle-Aged and Older Adult Patients

**DOI:** 10.3390/jfmk9040175

**Published:** 2024-09-26

**Authors:** Yuki Kurokawa, Satoshi Kato, Noriaki Yokogawa, Takaki Shimizu, Hidenori Matsubara, Tamon Kabata, Satoru Demura

**Affiliations:** Department of Orthopaedic Surgery, Kanazawa University, Takara-machi, Kanazawa 920-8641, Japan; kurokawa-yuki@med.kanazawa-u.ac.jp (Y.K.);

**Keywords:** respiratory function, trunk muscle, muscle strength

## Abstract

**Objectives:** Respiration plays an important function in sustaining life. The diaphragm is the primary muscle involved in respiration, and plays an important role in trunk stabilization. Although it has been reported that respiratory function is important for trunk muscle stability, the correlation between respiratory function and abdominal trunk muscle strength remains undetermined. This study aimed to clarify this correlation among middle-aged and older patients. **Methods:** This observational study included 398 patients scheduled for surgery for degenerative conditions of the lower extremities. Respiratory function was evaluated using forced vital capacity and forced expiratory volume in 1 s measured using spirometry. Each patient underwent a physical function test before surgery, which included the assessment of the abdominal trunk muscle strength, grip power, knee extensor strength, one-leg standing time, and gait speed. Correlations between abdominal trunk muscle strength, respiratory function, and physical function were evaluated. **Results:** Abdominal trunk muscle strength was significantly correlated with forced vital capacity, forced expiratory volume in 1 s, grip power, knee extensor strength, one-leg standing time, and gait speed. Multiple linear regression analyses revealed that sex, forced vital capacity, forced expiratory volume in 1 s, and knee extensor strength were significant factors associated with abdominal trunk muscle strength. **Conclusions:** In middle-aged and older patients, abdominal trunk muscle strength including that of the diaphragm, is associated with forced vital capacity and forced expiratory volume in 1 s.

## 1. Introduction

Breathing is a key function of the human body; it sustains life by providing oxygen for metabolic reactions and removing carbon dioxide, the generated byproduct [1]. The diaphragm is the primary muscle involved in active inspiration. Besides its respiratory function, the diaphragm also plays an important role in stabilizing the spine [2]. Diaphragmatic contraction contributes to spinal stability by increasing the intra-abdominal pressure (IAP); this is facilitated by a direct anatomical connection between the diaphragm and the lumbar spine [3,4]. In contrast, the abdominals are powerful expiratory muscles that play an important role in activities, such as forced expiration. The trunk stability has been shown to increase when the IAP is elevated by the contraction of the abdominal muscles [5]. Shirley et al. demonstrated that the stiffness of the spine is increased with both inspiratory and expiratory efforts [3]; therefore, the diaphragm and abdominals have an important role in respiratory function and trunk stability. 

**Figure 1:**
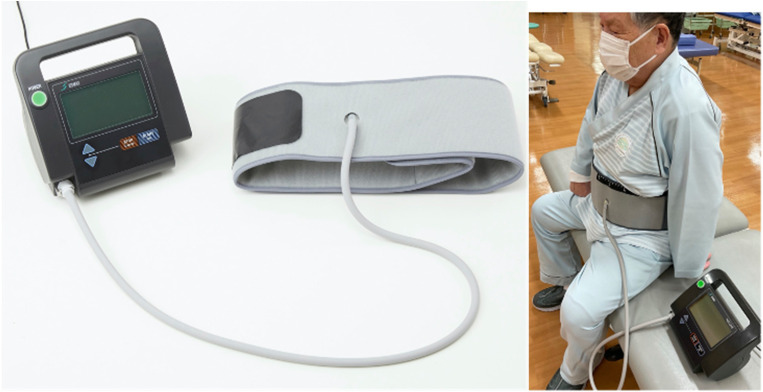
An innovative exercise device for the abdominal trunk muscles (RECORE). (**Left**) Photograph of the device. (**Right**) Photograph of a patient using the device to measure the ATMS. ATMS—abdominal trunk muscle strength.

Recently, an innovative exercise device for the abdominal trunk muscles (RECORE; Nippon Sigmax Co., Ltd., Shinjuku-ku, Tokyo, Japan; Figure 1) [6] was devised. This device makes it possible to strengthen the abdominal trunk muscles and to measure their strength. A previous study demonstrated that the strengthening exercises with the device activate the diaphragm, the abdominals, and the pelvic floor muscles, and that this device reliably measures strength exerted by the co-contraction of these muscles, called the abdominal trunk muscles, to increase the IAP and create a semirigid cylinder surrounding the abdominal cavity [7]. Thus, the abdominal trunk muscle strength (ATMS) measured using the device includes diaphragm muscle strength. We hypothesized that the ATMS, including that of the diaphragm, is associated with respiratory function. 

There is evidence of a relationship between respiratory function and the trunk muscles, including the diaphragm, in young healthy participants using ultrasound and electromyography. However, these studies involved a small number of patients, and few studies have been conducted in middle-aged and elder individuals [3,8,9,10]. Considering that muscle decline due to aging is greater in abdominal muscles than in other parts of the body [11], it is important to determine how the ATMS affects respiratory function in middle-aged and older adult patients. The purpose of this study was to clarify the relationship between respiratory function and ATMS, including that of the diaphragm, in middle-aged and older patients. 

## 2. Materials and Methods

### 2.1. Study Design

This cross-sectional study investigated the relationship between the ATMS evaluated by our original device and respiratory function. 

### 2.2. Patients

This study included 398 patients scheduled for surgery for degenerative diseases of the lower extremities at Kanazawa University Hospital who agreed to participate in pre-operative physical tests between December 2015 and July 2021. Written consent was obtained from all the patients before data collection. The degenerative diseases of the lower extremities included hip, knee, and foot and ankle joint diseases. The hip joint diseases included cases of avascular necrosis of the femoral head and osteoarthritis of the hip. These cases were scheduled for total hip arthroplasty. The diseases of the knee joint included knee osteoarthritis scheduled for total knee arthroplasty. The foot and ankle diseases included arthritis of the ankle and midfoot joints, as well as foot deformities treated with arthrodesis or corrective osteotomies of the ankle and foot. Patients with unilateral degenerative disease were included, whereas those scheduled for bilateral surgery were excluded. Patients who had previously undergone spinal surgery, those scheduled to undergo surgery for degenerative spinal disease, or those aged <39 years were excluded from this study. Spinal degenerative diseases have been reported to affect respiratory function [12,13]. Patients with a history of respiratory diseases that directly affect respiratory function, those with cardiac disease, and those with missing physical or laboratory data were also excluded from this study. We additionally excluded patients who had difficulty standing and walking on their own, and those who could not undergo measurement due to pain.

### 2.3. Description of the Device 

As previously outlined in detail [6,7], the exercise device has a design that may be considered similar to that of a sphygmomanometer, with an inflatable cuff and a built-in mechanical manometer to measure pressure. The device allows subjects to strengthen their abdominal trunk muscles and measure muscle strength in a seated position without moving the trunk or lumbar spine. For measurement, the cuff was placed around the subject’s abdomen and inflated. An electrically powered pump was used to inflate the cuff until sufficient resistance was encountered from the abdominal wall. Under the baseline pressure, the subject exerted the maximum amount of force by contracting the abdominal muscles. When measuring strength using the device, isometric muscle contraction occurred against the pressure from the cuff. The pressure in the cuff increased and reached a peak. The manometer reported the pressure generated by the subject (baseline pressure to peak pressure) to provide the value of the muscle strength (measured in kPa). The muscle strength value was defined as the ATMS. It was previously demonstrated that the device could measure the ATMS with excellent intra-rater reliability (intraclass correlation coefficients (ICC) = 0.95 a 95% confidence interval: 0.87–0.98) and inter-rater reliability (ICC = 0.99, confidence interval: 0.96–0.99) for muscle strength measurements [14]. During muscle-strengthening exercises using the device, the subject contracts the abdominals intermittently or continually under pressure from the cuff. This exercise is similar to the bracing exercise, which functions as a stabilization exercise [15]. The effectiveness of exercise therapy using this device has been reported previously. A 12-week program consisting of a total of 36 sessions, including 5-s muscle contractions followed by 5-s rests for 10 min at 50–80% of peak pressure measured by this device is effective [15].

### 2.4. Outcome Measures

The primary and secondary outcome measures used in this study were evaluated at the same time preoperatively. The primary outcome measures were the ATMS measured by the RECORE device and the respiratory functions were evaluated by the spirometry data. The ATMS was measured using previously established methods as described above. Muscle strength was measured twice, and the higher strength value was recorded. Spirometric data were obtained by clinical technicians according to the criteria established by the American Thoracic Society and European Respiratory Society for standardization [16]. The tests were performed at least thrice until successful, with the subjects in a relaxed sitting position. The respiratory function parameters analyzed were forced vital capacity (FVC) and forced expiratory volume in 1 s (FEV_1_). The FVC is the maximal volume of air exhaled with maximal forced effort from the maximal inspiration, and the FEV_1_ is the maximal volume of air exhaled in the first second of forced expiration following full inspiration [16]. The FEV_1_/FVC ratio (FEV_1%_) was also measured. 

The secondary outcome measures were grip power, knee extensor strength (KEMS), one-leg standing time (OLS), and gait speed. The grip power was measured using a Smedley-type hand dynamometer (Tsutsumi, Tokyo, Japan). Experienced medical staff instructed subjects to grip the dynamometer as strongly as possible at the handle. The right and left grip powers were measured once, and the higher value was recorded. The KEMS was measured using a handheld dynamometer (μTas F-1; Anima Corp., Tokyo, Japan). The patients were seated on elevated chairs with their knees flexed at 90° and their feet off the floor. With the dynamometer placed on the anterior surface of the leg, 10 cm proximal to the malleoli, the patients were instructed to push against the dynamometer by attempting to straighten their knees. This KEMS measurement method has previously demonstrated excellent reliability [17]. Before the evaluation, a sample was shown once and practiced with the subject, followed by a break of at least 30 s, after which the measurement was taken. The right and left KEMS were measured once, and the higher value was considered. A minimum of 30 s was given between each trial for recovery from fatigue. Additionally, the OLS was measured for each leg. The test spanned from when the subject raised their leg to when the leg was set back down on the floor, up to a maximum of 60 s. We performed the measurements twice, and the higher value was recorded. The 10-min gait time was the time taken to walk 10 m after walking 2 m. The gait speed (m/s) was calculated using the recorded time. A stopwatch device (Seiko, Chuo, Tokyo, Japan) was used to measure the time for the OLS and the gait speed. A five-point numeric rating scale (NRS) score (0 = no pain, 4 = severe pain) was also calculated for each instance of back, lumbar, and buttocks pain [18]. Additionally, the anthropometric measurements, including the body height, body weight, and body mass index (BMI), were obtained.

### 2.5. Statistical Analyses

Continuous variables are expressed as the mean ± standard deviation for parametric data, and *t*-tests were performed for comparisons. The distributions of the data were checked for normality using the Shapiro–Wilk test, and the homogeneity of the variances was checked using the Levene test. A Pearson correlation coefficient analysis was performed for the evaluation of the correlations between the ATMS and age, BMI, FVC, FEV_1_, FEV_1%_, grip power, KEMS, OLS, and gait speed. The correlation between the NRS score for back pain and other factors was evaluated using the Spearman correlation coefficient, because the NRS score was considered nonparametric data. A multiple linear regression analysis using a stepwise method was performed to identify the factors associated with the ATMS. We used Cook’s distance and centered leverage value to identify and treat outliers. Residual analyses were conducted to confirm the normality, linearity, equal variance, and independence. The correlation matrix, the normal probability plot (P-P) of the regression standardized residual, and scatterplots were used as parts of the analysis for checking the normality, linearity, homoscedasticity, and independence of the residuals. We confirmed that the dependent and independent variables were linear. The statistical significance was set at *p* < 0.05. The JMP version 11 software program (SAS Institute, Cary, NC, USA) was used to perform the statistical analyses.

The multiple linear regression required a minimum of 110 participants for the eight independent variables (FVC, FEV_1_, FEV_1%_, grip power, KEMS, OLS, gait speed, NRS for back pain) and three adjustment factors (age, sex, BMI), considering that a minimum of 10 cases was required per variable for a relatively stable model [19]. The statistical power of stepwise regression was calculated using the post hoc effect size (i.e., adjusted R^2^ of prediction line), α (0.05), the total sample size (398), and the number of predictors (10) using the G-power software (version 3.1., Franz Universitat, Kiel, Germany). 

## 3. Results

Table 1 lists the characteristics of the patients included in this study. The smoking rate was 28% (112/398); all patients had either already quit smoking or were being instructed to quit smoking to prepare for surgery.

No adverse events were observed while taking measurements using the device. The ATMS was significantly higher in male participants than in female participants (10.9 ± 4.6 kPa vs. 5.3 ± 3.4 kPa). Table 2 summarizes the mean values of the ATMS for the different age groups. There were significant differences in the ATMS among all the age groups (*p* < 0.01).

The values are depicted as mean ± standard deviations. A *p*-value of <0.05 was considered statistically significant. Pearson’s correlation coefficient (r) indicates the post hoc effect size. 

Table 3 presents the correlations between the ATMS and other factors in all the patients. The ATMS measured by the device significantly correlated with the FVC (r = 0.54, *p* < 0.01), FEV_1_ (r = 0.46, *p* < 0.01), FEV_1%_ (r = 0.14, *p* < 0.01), grip power (r = 0.54, *p* < 0.01), KEMS (r = 0.55, *p* < 0.01), OLS (r = 0.26, *p* < 0.01), and gait speed (r = 0.32, *p* < 0.01). However, there was no correlation between the BMI and NRS scores for back pain. Similar to the ATMS, the grip power and KEMS were correlated with the FVC (grip power, r = 0.71, *p* < 0.01; KEMS, r = 0.57, *p* < 0.01), FEV_1_ (grip power, r = 0.66, *p* < 0.01; KEMS, r = 0.51, *p* < 0.01), and FEV_1%_ (grip power, r = 0.13, *p* < 0.01; KEMS, r = 0.10, *p* < 0.01). The FVC and FEV_1_ also correlated with the OLS (FVC, r = 0.32, *p* < 0.01; FEV_1_, r = 0.33, *p* < 0.01) and gait speed (FVC, r = 0.30, *p* < 0.01; FEV_1_, r = 0.27, *p* < 0.01). Considering the sex, the ATMS significantly correlated with the FVC (mele: r = 0.39, *p* < 0.01; female: r = 0.29, *p* < 0.01), FEV_1_ (female: r = 0.24, *p* < 0.01), grip power (male: r = 0.45, *p* < 0.01; female: r = 0.26, *p* < 0.01), KEMS (male: r = 0.45, *p* < 0.01; female: r = 0.45, *p* < 0.01), OLS (male: r = 0.39, *p* < 0.01; female: r = 0.21, *p* < 0.01), and gait speed (male: r = 0.48, *p* < 0.01; female: r = 0.27, *p* < 0.01). The multiple linear regression analysis revealed that the sex (female: β = −0.18, *p* < 0.01), FVC (β = 0.34, *p* < 0.01), FEV_1_ (β = 0.19, *p* < 0.05), and KEMS (β = 0.26, *p* < 0.01) were significantly associated with the ATMS, describing 41% of the adjusted R^2^ value (Table 4). The statistical power of the stepwise regression was 1.00.

## 4. Discussion

The present study aimed to assess the relationship between the respiratory function and the ATMS, including that of the diaphragm in middle-aged and older patients. We found that the ATMS was associated with the respiratory function, as measured based on the FVC and the FEV_1_. Furthermore, in the univariate analyses, the FVC and FEV_1_ were correlated with the grip power, KEMS, OLS, and gait speed. Previous studies have demonstrated that extremity muscle strength, particularly hand grip strength, is significantly correlated with pulmonary function in older people [20,21]. Another study revealed that the deterioration of respiratory function was closely related to the deterioration of balance maintenance [4]. Additionally, gait speed is reported to be associated with pulmonary function [22,23]. Herein, the ATMS was also correlated with the grip power, OLS, and gait speed in the univariate analyses. A systematic review has reported that weakness in trunk muscle strength was associated with poor balance and functional performance in older adults [24]. Furthermore, it was previously demonstrated that weak ATMS, as measured using this device, was associated with a decline in balance and function, and an increased risk of falling [25]. Our results support that of previous reports.

According to the multivariate analysis, sex, FVC, FEV_1_, and KEMS were significant factors associated with ATMS. Respiratory function tests are commonly performed during routine medical check-ups and prior to surgery. Of the respiratory functional tests, FVC and FEV_1_ are important indicators used for diagnosing the presence of an obstructive ventilatory disorder [26]. The results of the present study suggest that FVC and FEV_1_, which are important factors in respiratory function, are related to ATMS, including that of the diaphragm, in middle-aged and older patients. Similar to our findings, Yüksel et al. reported positive relationships between respiratory function, including FVC and FEV_1_, and trunk endurance tests, including prone bridge and flexor endurance tests, among young healthy individuals [10]. A previous study demonstrated that the abdominal rectus, diaphragm, internal oblique, external oblique, transverse abdominis, and levator ani muscles were significantly activated during strengthening exercises with the device [7]. The core can be described as a muscular box with the abdominals at the front and sides, the paraspinal muscles at the back, the diaphragm at the roof, and the pelvic floor at the bottom of the box. These muscles work together to generate abdominal pressure and maintain spinal stability [27]. The abdominal contraction maneuver while measuring the ATMS produced by the device is similar to abdominal bracing [28] and produces a coordinated contraction of the deep and superficial core muscles, primarily at the anterolateral aspect and the upper and lower sides of the core muscular box [7].

The diaphragm is the principal muscle involved in inspiration [29], and contributes to trunk stiffness and postural control [30]. The rectus abdominis, transverses abdominis, internal oblique, and external oblique muscles are the most powerful muscles involved in expiration [31]. Although the transverse abdominis has the lowest threshold for the respiratory activity of the ATMS [32], it is thought to be important for the control of the intersegmental stiffness of the spine via the increased IAP or the tension in the thoracolumbar fascia [33]. Therefore, the abdominal trunk muscles activated by the device play an important role in both respiratory function and trunk stability. Many studies have reported a relationship between respiratory function and postural control, lower back pain, and trunk stability. A previous study investigated the contribution of the diaphragm to postural control using electromyography. Their results indicated that the activation of the diaphragm may assist in the mechanical stabilization of the trunk, in addition to the maintenance of ventilation [34]. Moreover, in subjects with low back pain, magnetic resonance imaging showed a reduction in diaphragm excursion [35]. However, the sample sizes of these studies were relatively small. To our knowledge, no studies have measured trunk muscle strength, including the strength of the diaphragm, and verified its relationship with respiratory function. Previously, it has been demonstrated that no participants experienced adverse events, including the worsening of back pain, the onset of pain, or abdominal discomfort, during strength testing with this device [6,7,25,36]. The strength evaluation with this device does not cause stress and/or pain in the lumbar spine or extremities; therefore, they have spinal deformities, and experience severe pain. We were able to collect data from a large number of middle-aged and older patients who were scheduled to undergo surgery for degenerative diseases of the lower extremities because measurements with this device were easier and more convenient than that with conventional methods for muscle strength measurement, such as isometric and isokinetic tests using the Cybex [37] and Biodex [38] Systems. 

In multivariate analyses, sex and KEMS were also significantly associated with ATMS. Additionally, there were significant sex-related differences in the ATMS across all the age groups. It is known that the abdominal core muscles are consistently activated before any limb movements [39]. Therefore, a function of these muscles is to assist in the production of force or power in the limbs during kinetic chain activities [40]. Many studies have examined the effect of core stabilization training on muscle strength and the performance of various activities, such as leg presses [41], squats [42,43], and jump performances [44]. However, the results of these studies are inconsistent because of the different modes of exercise and muscle strength measurement techniques used. The relationship between ATMS, including diaphragm strength, and lower limb muscle strength has not been previously reported. Our study indicates that ATMS, including diaphragm strength, is associated with KEMS. This result supports the conclusion of previous studies that trunk stabilization exercises affect lower extremity muscle strength [45,46].

The dual functions of the diaphragm (ventilation and postural control) are performed simultaneously. The increased demand for one of its functions (an inspiratory loading task) will inevitably diminish the other function and often reduces the ability of the respiratory muscles to perform their postural duties [4]. A previous study demonstrated that a diaphragm-strengthening training program is a viable approach toward increasing the thickness of the diaphragm and other stabilizer muscles of the lumbar spine [47]. However, the training methods used in previous studies varied, and the most effective training methods for enhancing respiratory function and trunk stability have not been established. 

The results of this study indicate that the ATMS, including that of the diaphragm, is associated with respiratory function. We speculate that respiratory function is simultaneously important in strengthening trunk muscle strength as physical therapy for middle-aged and older patients, which may also aid in training patients with impaired respiratory function. However, middle-aged and older adults, especially the elderly, may have difficulty performing general trunk muscle strengthening exercises due to pain and reduced flexibility. For such patients, exercising while controlling their breathing enhances the difficulty.

Exercise using the device is more easily accessible to elderly patients; therefore, the device has the potential to simultaneously enhance trunk stability and respiratory function and could be useful for the treatment of middle-aged and elderly patients with respiratory and trunk dysfunctions. We hypothesize that respiratory function and core muscle strength training may be more easily accomplished than high-level core training involving controlled/focused breathing. Further research is needed to test this hypothesis.

The present study had some limitations. This study included patients with degenerative diseases of the lower extremities, which may have influenced the results. Therefore, studies using healthy subjects are warranted in the future. Moreover, the study cohort included more women than men. This could be because the study population included a large number of patients with osteoarthritis of the hip and knee, which have been reported to have a higher prevalence among women [48,49]. Despite these limitations, this study was able to establish the relationship between respiratory function and the ATMS, including the strength of the diaphragm, which had not been reported previously.

## 5. Conclusions

The ATMS, including the strength of the diaphragm, was associated with the respiratory function measured using FVC and FEV_1_ in middle-aged and older patients. 

## Figures and Tables

**Table 1:** Patient characteristics.

Characteristics	
No. of patients	398
Sex	Male (68)
	Female (330)
Age (years), mean ± SD [range]	65.6 ± 9.5 [41–88]
Height (cm), mean ± SD [range]	154.9 ± 8.4 [129.8–186.2]
Weight (kg), mean ± SD [range]	58.2 ± 11.4 [31.6–104.6]
Body mass index (kg/cm^2^), mean ± SD [range]	24.2 ± 4.2 [14.1–43.5]
FVC, mean ± SD [range]	2.99 ± 0.74 [1.57–5.93]
FEV_1_, mean ± SD [range]	2.21 ± 0.55 [1.1–4.54]
FEV_1%_, mean ± SD [range]	73.8 ± 97.5 [48.2–97.5]
Degenerative diseases of the lower extremities treated with subjects	Hip joint diseases (251)
	Knee joint diseases (81)
	Foot and ankle joint diseases (66)

**Table 2:** The mean values of the abdominal trunk muscle strength (kPa) measured by the novel device.

Age Groups (Years) (Number of Male/Female)	Male (n = 68)	Female (n = 328)	*p*-Value	r
40–49 (5/20)	12.8 ± 3.7	5.8 ± 4.0	<0.01	0.60
50–59 (17/66)	12.9 ± 6.3	5.5 ±3.7	<0.01	0.57
60–69 (22/118)	10.7 ± 3.3	5.3 ± 3.4	<0.01	0.51
70–79 (19/105)	9.5 ± 3.9	5.2 ± 3.2	<0.01	0.41
80–89 (5/19)	9.2 ± 3.7	3.4 ± 2.4	<0.01	0.67

**Table 3:** The correlation coefficients between abdominal trunk muscle strength and other factors.

	ATMS	Age	BMI	FVC	FEV_1_	FEV_1%_	Grip Power	KEMS	OLS	Gait Speed	NRS for LBP
ATMS		−0.15 **	−0.34	0.54 **	0.46 **	0.14 **	0.54 **	0.55 **	0.27 **	0.32 **	−0.09
Age	−0.15 **		0.08	−0.34 **	−0.40 **	−0.12 **	−0.38 **	−0.22 **	−0.46 **	−0.26 **	0.01
BMI	−0.02	0.08		−0.03	−0.02	−0.01	0.07	0.13 **	−0.22 **	−0.14 **	0.07
FVC	0.54 **	−0.33 **	−0.03		0.91 **	−0.23 **	0.71	0.57 **	0.32 **	0.30 **	−0.11 *
FEV_1_	0.46 **	−0.40 **	−0.02	0.91 **		0.06	0.66 **	0.51 **	0.33 **	0.27 **	−0.09
FEV_1%_	−0.14 **	−0.12 **	−0.01	−0.23 **	0.06		−0.13 **	−0.10 **	0.04	−0.02	0.05
Grip power	0.54 **	−0.38 **	0.07	0.71 **	0.66 **	0.13 **		0.65 **	0.43 **	0.39 **	−0.01
KEMS	0.55 **	−0.22 **	0.13 **	0.57 **	0.51 **	0.10 **	0.65 **		0.36 **	0.41 **	−0.10 *
OLS	0.27 **	−0.46 **	−0.22 **	0.32 **	0.33 **	0.04	0.43 **	0.36 **		0.41 **	−0.01
Gait speed	0.32 **	−0.26 **	−0.14 **	0.30 **	0.27 **	−0.02	0.39 **	0.41 **	0.41 **		−0.12 **
NRS for LBP	−0.09	0.009	0.07	−0.11	−0.09	0.05	−0.01	−0.10 *	−0.01	−0.12 *	

ATMS—abdominal trunk muscle strength; BMI—body mass index; FEV_1_—forced expiratory volume in 1 s; FVC—forced vital capacity; FEV_1%_—FEV_1_/FVC ratio; KEMS—knee extensor muscle strength; OLS—one-leg standing time; NRS for LBP—numerical rating scale for low back pain. A *p* value of <0.05 was considered statistically significant. Asterisk indicates statistically significant data (* *p* < 0.05, ** *p* < 0.01).

**Table 4:** Stepwise multiple linear regression analysis for the factors associated with abdominal trunk muscle strength.

Independent Variable	Standardized β	*p*-Value	VIF
Sex (Female)	−0.18	<0.01	2.13
BMI	−0.06	0.12	1.12
FVC	0.34	<0.01	7.61
FEV_1_	0.19	0.04	6.14
Grip power	0.10	0.12	2.83
Strength of the knee extensor	0.26	<0.01	2.10
Gait speed	0.09	0.06	1.39

Variables extracted after stepwise variable selection are presented. R^2^ = 0.42, adjusted R^2^ = 0.41, *p* < 0.01. BMI—body mass index; FEV_1_—forced expiratory volume in 1 s; FVC—forced vital capacity; VIF—variance inflation factor.

## Data Availability

The data presented in this study are available on request to the corresponding author. The data are not publicly available due to personal protection.
